# Rapid response events with multiple triggers are associated with poor outcomes in children

**DOI:** 10.3389/fped.2023.1208873

**Published:** 2023-06-14

**Authors:** Erin M. Kritz, Jenilea K. Thomas, Nawara S. Alawa, Elit B. Hadad, Danielle M. Guffey, Aarti C. Bavare

**Affiliations:** ^1^ Department of Pediatric Critical Care, Baylor College of Medicine, Houston, TX, United States; ^2^ Department of Pediatrics, Texas Children’s Hospital, Houston, TX, United States

**Keywords:** pediatric outcomes, rapid response, pediatric ICU, critical deterioration, cardiopulmonary arrest (cpa), triggers

## Abstract

**Objective:**

We describe the characteristics and outcomes of pediatric rapid response team (RRT) events within a single institution, categorized by reason for RRT activation (RRT triggers). We hypothesized that events with multiple triggers are associated with worse outcomes.

**Patients and Methods:**

Retrospective 3-year study at a high-volume tertiary academic pediatric hospital. We included all patients with index RRT events during the study period.

**Results:**

Association of patient and RRT event characteristics with outcomes including transfers to ICU, need for advanced cardiopulmonary support, ICU and hospital length of stay (LOS), and mortality were studied. We reviewed 2,267 RRT events from 2,088 patients. Most (59%) were males with a median age of 2 years and 57% had complex chronic conditions. RRT triggers were: respiratory (36%) and multiple (35%). Transfer to the ICU occurred after 1,468 events (70%). Median hospital and ICU LOS were 11 and 1 days. Need for advanced cardiopulmonary support was noted in 291 events (14%). Overall mortality was 85 (4.1%), with 61 (2.9%) of patients having cardiopulmonary arrest (CPA). Multiple RRT trigger events were associated with transfer to the ICU (559 events; OR 1.48; *p* < 0.001), need for advanced cardiopulmonary support (134 events; OR 1.68; *p* < 0.001), CPA (34 events; OR 2.36; *p* = 0.001), and longer ICU LOS (2 vs. 1 days; *p* < 0.001). All categories of triggers have lower odds of need for advanced cardiopulmonary support than multiple triggers (OR 1.73; *p* < 0.001).

**Conclusions:**

RRT events with multiple triggers were associated with cardiopulmonary arrest, transfer to ICU, need for cardiopulmonary support, and longer ICU LOS. Knowledge of these associations can guide clinical decisions, care planning, and resource allocation.

## Introduction

Rapid response teams (RRT) are widely implemented and have demonstrated a positive impact on patient safety and outcomes (1–7). The goal of RRTs is to manage deteriorating hospitalized patients to decrease out of ICU cardiac arrests and prevent morbidity and mortality (8–10). It is important to identify patient and RRT event factors which may signal need for transfer to a higher level of care and further critical care interventions (11–13).

Adult literature suggests that certain signs and symptoms of clinical instability that trigger RRT events are associated with worse patient outcomes. The nature and number of triggers can guide appropriate resource allocation to prevent further deterioration and adverse outcomes (14–18). Most adult studies classify RRT triggers under the categories of respiratory, cardiac, neurological, medical, or other/unknown, with respiratory and cardiac triggers being most common (19). Respiratory triggers have been described as predictive of mortality in pediatric and adult inpatients (19–21). Adult studies have found an increase in in-hospital mortality with the number of simultaneous RRT triggers (22). This work expands the pediatric literature to describe and evaluate multiple trigger RRT events with patient outcomes.

We reviewed the triggers of RRT events within a single hospitalization at a large academic pediatric hospital. We hypothesized that RRT events due to multiple triggers are associated with worse outcomes. Early identification of patients at risk for further deterioration at the time of RRT event can guide early resuscitation efforts, aid clinical decision and disposition determination, and improve communication between ICU and acute care providers. For patients with chronic conditions, this can facilitate early discussion regarding disease prognostication and resuscitation status.

## Methods

### Study design and setting

We conducted a retrospective study of index RRT events over a 3 year-period to compare patient and RRT event characteristics, interventions, and outcomes. The institutional review board approved this study. Our hospital is a large academic pediatric hospital with two community campuses with around 1,000 RR events per year. Our institution has a well-established RRT policy in which the RRT team can be activated by any concerned hospital provider or family member through a centralized number. RRT teams are composed of ICU staff who respond promptly (within 15 min per institutional policy) to RRT activations. Activation criteria (triggers) include any form of respiratory, cardiac, or neurologic decompensation of hospitalized patients who are outside ICU settings. There are no automatic criteria for activation based on patient acuity scores or vital sign parameters. ICU admission is not required for initiation of high-flow nasal cannula within a maximum flow per age, for continuous albuterol therapy, or for patients on home noninvasive ventilation settings. Our central and community campuses have ICUs and follow the same RRT procedures. Further details on our institution's RRT and code team composition, triggers, and activation process are outlined in Supplementary Table S1.

### Data collection, variables, and definitions

We collected data from RRT administrative database, electronic medical records, and the Pediatric Health Information System database. Our institutional RRT administrative database includes patient and event details for all RRT events. Patient demographic information was collected as well as clinical data, including primary diagnosis, admission and discharge dates, and primary service. RRT event details and outcomes collected were date and time of event, time to and duration of response, interventions required, trigger(s) for activation, transfer to ICU, additional cardiopulmonary support required within 24 h of transfer, mortality, and cardiopulmonary arrest at time of RRT event. Triggers were categorized and entered into the medical record by the RRT team based upon information provided during handoff by the activating team and assessment of clinical signs and symptoms present during RRT event. Trigger categories are respiratory, cardiac, neurologic, and other based upon standardized definitions at our institution. Events with triggers in more than one organ system were noted as multiple triggers. Chart review by an independent investigator was performed to ensure the original trigger categorization held true. Standardized trigger definitions at our institution are as follows:

**Table:** 

RRT Activation Triggers	Details
Respiratory	Decreased oxygen saturation from baseline
	Increased work of breathing
	Tachypnea
	New onset of difficulty breathing
	Bleeding into the airway
	Respiratory depression
Hemodynamic	Change in heart rate (tachycardia or bradycardia)
	Hypotension
	Concerning change in perfusion
Neurologic	Change in mental status
	Seizure
Other	Staff concern
	Family worry
	Difficult to control pain
	Concerning decrease in urine output in preceding 12 h
Multiple	Trigger from 1 or more above categories

Primary diagnosis was used to categorize patients based upon the principal organ system involved. Consistent with the literature, patients were classified as having complex chronic conditions if they had a “medical condition that can be reasonably expected to last at least 12 months (unless death intervenes) and to involve either several different organ systems or one organ system severely enough to require specialty pediatric care” (23, 24). This included patients with chronic conditions, history of prematurity, organ transplant, and/or technological dependence.

Outcome variables reviewed included acute respiratory compromise (ARC, need for bag-mask ventilation or endotracheal intubation) or cardiopulmonary arrest (CPA) during RRT event, transfer to higher level of care, need for advanced cardiopulmonary support (defined as need for mechanical ventilation and/or hemodynamic support within 24 h of RRT event), hospital and ICU length of stay (LOS), and in-hospital mortality.

### Statistical analyses

Patient and RRT characteristics and outcomes are summarized using mean with standard deviation, median with interquartile range (IQR), and frequency with percentages. Characteristics and outcomes are compared by triggers using quantile regression, Chi-square test and Fisher's exact test. Simple and multivariable logistic regression was used to assess the association between variables age, gender, primary organ system, complex chronic conditions, RRT trigger, and main campus location and the odds of requiring advanced cardiopulmonary support (need for mechanical ventilation and/or hemodynamic support within 24 h of RRT activation) and odds of mortality. Simple and multivariable quantile regression was used to assess the association between variables age, gender, primary organ system, complex chronic conditions, RRT trigger, and main campus location and the median hospital and ICU LOS. All models adjust for the same factors. The Hosmer-Lemeshow test assessed the goodness of fit of the model and multicollinearity was assessed with the variance inflation factor. There were not goodness of fit or multicollinearity issues found. For simplicity we are presenting the index RRT and index admission per patient to avoid correlated data issues. All analyses are performed using Stata 15.0 (Stata Corp, College Station, TX).

## Results

### Patient and RR event characteristics

We reviewed a total of 2,267 RRT events in 2,088 patients and included the index 2,088 RRT events in our analysis.Triggers for RRT activation were primarily respiratory [758 (36.3%)] and multiple [739 (35.4%)], with the remainder being cardiac [236 (11.3%)], neuro [218 (10.4%)], and other [137 (6.6%)]. Table 1 presents the patient characteristics by RR triggers.

**Table 1:** Patient characteristics by RRT trigger.

	Respiratory (*n* = 758)	Cardiac (*n* = 236)	Neuro (*n* = 218)	Other (*n* = 137)	Multiple (*n* = 739)	Total Population (*n* = 2,088)	*P* value
Median age at event (yr) (IQR)	1.2 (0.3, 4.0)	8 (1.5,14.0)	9 (3.0,14.0)	6 (1.4, 13.0)	1.8 (0.4, 8.0)	2 (0.5, 10)	< 0.001
Male, *n*(%)	474 (62.5)	129 (54.7)	106 (48.6)	73 (53.3)	446 (60.4)	1,228 (58.8)	0.001
Complex Chronic Condition, *n*(%)	385 (50.9)	154 (65.5)	161 (74.2)	88 (64.7)	389 (52.7)	1,177 (56.5)	< 0.001
Single RR Event During Index Admission, *n*(%)	707 (93.3)	218 (92.4)	190 (87.2)	132 (96.4)	692 (93.6)	1,939 (92.9)	0.012
Central Campus Location, *n*(%)	600 (79.2)	207 (87.7)	186 (85.7)	109 (79.6)	586 (79.4)	1,688 (80.9)	0.011

RRT, Rapid Response Team; ICU, Intensive care unit; LOS, length of stay; IQR, interquartile range.

Variables expressed as number (percentage) or median (interquartile range) as appropriate.

*P*-values calculated by quantile regression, Chi-square test, or Fisher's exact test for statistical difference between any trigger groups.

### Outcomes

Transfer to the ICU occurred after 1,468 events (70%). Median hospital LOS was 11 days (IQR 9–12 days) and median ICU LOS was 1 day (IQR 0–1 days). Need for advanced cardiopulmonary support within 24 h was noted in 291 events (14%): mechanical ventilation [196 (67%)], vasoactive medications [34 (12%)], and mixed cardiac and respiratory support [61 (21%)]. Hospital mortality for the cohort was 4.1% (85), with 61 (2.9%) of patients having CPA during the event.

### Outcomes analyses

**Figure 1:**
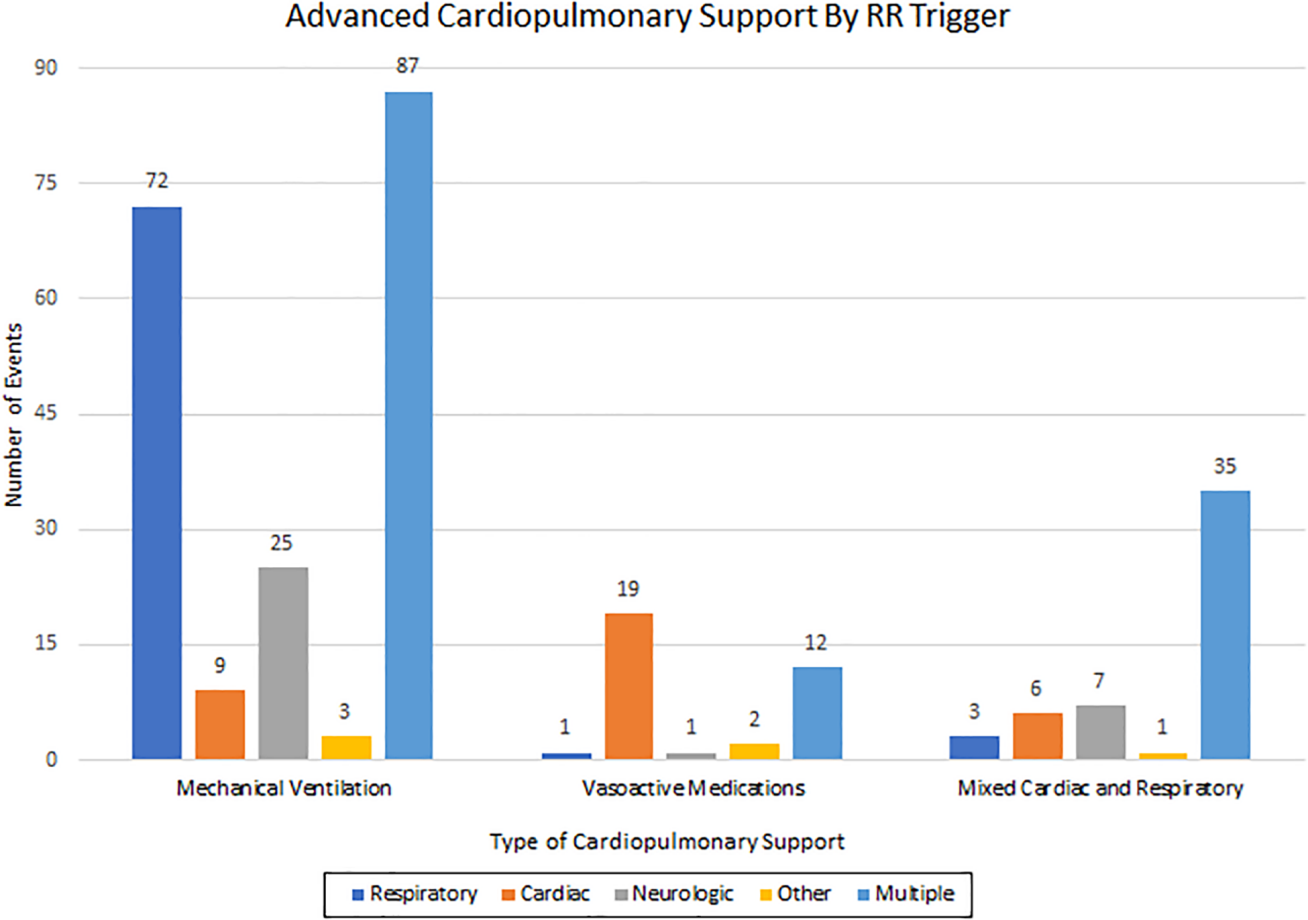
Type of advanced cardiopulmonary support by trigger, *p* = <0.001. Type of advanced cardiopulmonary support required depicted by RRT trigger. RRT, rapid response team.

Multiple trigger RRT events had higher rate of transfer to ICU (559 patient events; OR 1.48; *p* < 0.001) and higher rate of CPA (34 events, OR 2.36; *p* = 0.001) than RRT events with any other types of triggers. Hospital LOS was longer for patients with chronic medical conditions. Type of trigger, however, was not associated with length of stay after adjusting for confounders of age, gender, primary organ system, and chronic condition. ICU LOS is longest in multiple trigger events (2 vs. 1 days; IQR 0–5 d; *p* < 0.001). All categories of triggers have lower odds of need for advanced cardiopulmonary support than multiple triggers (unadjusted OR 1.73; CI: 1.29–2.32; *p* < 0.001), Figure 1. After adjusting for confounders, multiple triggers were associated with higher odds for advanced cardiopulmonary support compared to other triggers: respiratory (adjusted OR, 1.68; CI: 1.25–2.27; p = 0.001), cardiac (adjusted OR: 1.49; CI: 0.97–2.29; p < 0.001), neuro (adjusted OR: 1.69; CI: 1.07–2.68; p = 0.03), and other triggers (adjusted OR: 5.13; CI: 2.19–12.01; p < 0.001). Patients with chronic conditions were found to have higher odds of mortality (OR, 6.7; CI 2.34–19.39; *p* < 0.001). After adjusting for confounders, trigger was not independently associated with mortality. Table 2 details RRT event outcomes by triggers.

**Table 2:** Patient Outcomes by RR Trigger.

	Respiratory (*n* = 758)	Cardiac (*n* = 236)	Neurologic (*n* = 218)	Other (*n* = 137)	Multiple (*n* = 739)	*P* value
Median ICU LOS (days) (IQR)	1 (0.0,4.0)	1 (0.0,4.0)	1 (0.0,4.0)	1 (0.0,3.0)	2 (0.0, 5.0)	<0.001
Median Hospital LOS (days) (IQR)	7 (4.0, 17.0)	12 (6.0, 30.0)	12 (5.0, 27.0)	11 (5.0, 24.0)	9 (5.0, 23.0)	<0.001
Mortality, *n*(%)	27 (3.6)	16 (6.8)	7 (3.2)	3 (2.2)	32 (4.3)	0.189
Cardiopulmonary Arrest, *n*(%)	17 (2.2)	2 (0.8)	8 (3.7)	0 (0.0)	34 (4.6)	0.001
Transferred to ICU, *n*(%)	534 (74.1)	162 (72.0)	140 (67.0)	73 (57.9)	559 (78.3)	<0.001

RR, Rapid Response, ICU, Intensive care unit, LOS, length of stay, IQR, interquartile range.

Variables expressed as number (percentage) or median (interquartile range) as appropriate.

*P*-values calculated by quantile regression, Chi-square test, or Fisher’s exact test for statistical difference between any trigger groups.

## Discussion

There is limited pediatric data available regarding RRT triggers and association with outcomes. Identification of at-risk patients often includes the use of triggers which are informed by aggregate pediatric early warning scores, monitored vital signs, flagging of diagnostic risk factors, as well as medical team and parental concern regarding clinical status. To our knowledge, this is the largest cohort in pediatric patients used to assess the association of RRT triggers with outcomes (25–31). Our results support our hypothesis that RRT trigger was associated with several outcomes. Children with multiple triggers for RRT events had longer ICU LOS, higher rates of cardiopulmonary arrest, and need for advanced cardiopulmonary support, which may signal a sicker patient who requires advanced ICU interventions and longer ICU stay. As initiation and use of NIPPV can be somewhat subjective and dependent on specific practices to each center, we did a separate sub analysis of patients who required mechanical ventilation and/or hemodynamic support; and indeed, patients with multiple-trigger RRT events did more often require this type of support. We felt these represented more objective ICU-level interventions which could be interpreted and applied to a variety of centers. Although trigger type was not associated with mortality, the association with other significant outcomes could impact clinical decisions, care planning, and resource allocation.

As trigger is known at the time of RRT event, this may offer a means of prognostication and allocation of resources. Patients with multiple triggers may benefit from more thorough evaluation by the ICU team with a lower threshold to transfer to higher level of care. This categorization may also aid expectations regarding length of stay and level of monitoring in the ICU or acute care ward, as well as higher vigilance by the clinical team for signs of further deterioration.

The main limitations of this study relate to its retrospective nature. Outcomes data for a few patients was either incomplete or unavailable. Given the overall rarity of catastrophic outcomes in the pediatric population, it was difficult to demonstrate association with some outcomes. Finally, this work was conducted at a single center with processes and procedures that may be unique to our institution, thereby limiting generalizability. Nevertheless, we believe this is an expanded approach to RRT event analysis in pediatric patients and offers potential for prognostication regarding outcomes at the time of event.

## Conclusion

We confirmed that RRT events with multiple triggers were associated with increased ICU length of stay, increased frequency of cardiopulmonary arrest outside of the ICU, and need for a higher level of care. Regular review of RRT events and outcomes can inform a more targeted use of triggers to better prognosticate patient outcomes and effective allocation of resources.

## Data Availability

The raw data supporting the conclusions of this article will be made available by the authors, without undue reservation.
