# Supplementary material for: Rapid response events with multiple triggers are associated with poor outcomes in children
Source: Front Pediatr. 2023 Jun 14;11:1208873. doi: 10.3389/fped.2023.1208873 (PMC10303937; doi:10.3389/fped.2023.1208873)
Supplement: Supplementary file 1 [file Table1.docx]

Supplemental Table 1: Institutional Rapid Response System

| Component | Details |
| --- | --- |
| RR Team Personnel | Critical care fellow or APP  Critical care RN  Critical care RT  Pediatric resident  Nurse house supervisor |
| Code Event Personnel | Critical care fellow or APP  Critical care RN  Critical care RT  Pediatric resident  House supervisor  Critical care attending physician  Pharmacist  Chaplain |
| RR Activating Personnel | Clinical team: RNs, RTs, residents, fellows, attending providers  Family members |
| RR Triggers | **Respiratory:** tachypnea, increased work of breathing, decreased oxygen saturation, respiratory depression  **Hemodynamic:** bradycardia, tachycardia, hypotension, hypertension, chest pain, change in perfusion  **Neurologic:** change in mental status, seizure, suspected stroke  **Other:** staff or family concern, uncontrolled pain, uncontrolled bleeding |
| Code Triggers | Ineffective respiration  Ineffective circulation  Altered consciousness |

Supplemental table 1: Components and details of our institutional rapid response system.

RR = rapid response, APP = advanced practice provider, RN = registered nurse, RT = respiratory therapist.
